# Astrocytic Accumulation at the Choroid Plexus Attachment Region

**DOI:** 10.1007/s10571-026-01742-6

**Published:** 2026-05-18

**Authors:** Katerina Manzhula, Theresa Greiner, Louise Baumann, Hannes Kaddatz, Vladislav Yakimov, Marcus Frank, Björn Schneider, Markus Kipp, Sarah Joost

**Affiliations:** 1 Institute of Anatomy, Rostock University Medical Center, Rostock, Germany; 2 Department of Neurology, Rostock University Medical Center, Rostock, Germany; 3 Centre for Transdisciplinary Neurosciences Rostock (CTNR), Rostock University Medical Center, Rostock, Germany; 4 Institute of Anatomy II, Faculty of Medicine, LMU Munich, Munich, Germany; 5 Department of Psychiatry and Psychotherapy, LMU University Hospital, LMU Munich, Munich, Germany; 6 International Max Planck Research School for Translational Psychiatry (IMPRS-TP), Munich, Germany; 7 Electron Microscopy Centre, Rostock University Medical Center, Rostock, Germany; 8 Department of Life, Light and Matter, Rostock University, Rostock, Germany; 9 Institute of Pathology, Rostock University Medical Center, Rostock, Germany

**Keywords:** Choroid plexus, Neuroinflammation, Glia limitans, Barrier, Astrocyte, Basal lamina

## Abstract

Immune cell entry into the central nervous system (CNS) under neuroinflammatory conditions is commonly discussed in relation to established barrier interfaces, including the blood–brain barrier and the choroid plexus epithelium. In addition, an alternative anatomical route at the choroid plexus attachment region has been proposed, yet its structural organization remains poorly defined. Here, we provide a detailed descriptive analysis of the choroid plexus attachment region and identify glial structures reminiscent of a glia limitans. We analyzed the morphology of the choroid plexus attachment region in control mice using immunohistochemical and ultrastructural techniques. Immune cell accumulation under neuroinflammatory conditions was examined in the CupEAE mouse model, and human post-mortem sections were analyzed for comparative insights. Our results show that the murine choroid plexus attachment region contains an astrocytic accumulation in close proximity to a continuous basal lamina facing the choroid plexus stroma. Ultrastructural analyses indicate the presence of components associated with junctional complexes. In the CupEAE model, CD3-positive immune cells were more frequently observed in the choroid plexus base than in the brain parenchyma adjacent to the attachment region. In humans, choroid plexus attachment regions also exhibit astrocytic accumulation, though with a different basal lamina pattern. These results underline the complex organization of the choroid plexus attachment region. This anatomical description might be the basis for further studies to evaluate the relevance of the choroid plexus attachment region in immune cell migration to the brain parenchyma.

**Supplementary Information:**

The online version contains supplementary material available at 10.1007/s10571-026-01742-6.

## Introduction

The central nervous system (CNS) is traditionally regarded as an immune-privileged environment. However, in neuroinflammatory conditions such as multiple sclerosis, peripheral immune cells can infiltrate the CNS parenchyma. While the molecular mechanisms governing immune cell trafficking across the brain vasculature and leptomeninges have been studied extensively, the anatomical and cellular pathways facilitating immune cell entry at the level of the choroid plexus remain less well defined (Engelhardt et al. 2017).

The choroid plexus, located in all four ventricles of the brain, is a highly vascularized structure responsible for the production of cerebrospinal fluid (CSF). In addition to its role in CSF production, the choroid plexus functions as a dynamic signaling interface between the CNS and the CSF compartment, secreting a variety of factors that influence neural and immune processes within the brain (Lehtinen et al. 2011; Lun et al. 2015). The epithelium of the choroid plexus forms the blood-CSF-barrier through apically located tight junctions (Kaur et al. 2016) and therefore isolates the CSF-filled ventricle from the choroid plexus stroma. The stroma of the choroid plexus is composed of capillary-rich connective tissue, which is continuous with the leptomeninx (arachnoid and pia mater) (Ghersi-Egea et al. 2018). Unlike the restrictive blood-brain barrier, the fenestrated endothelium of the choroidal blood vessels (Cornford et al. 1998) permits immune cell entry into the choroid plexus stroma.

Under pathological conditions, immune cells within the choroid plexus stroma have been shown to traverse the blood-CSF barrier, enter the ventricular space, and subsequently cross the ependymal cell layer (the epithelium of the ventricular system) to infiltrate the CNS parenchyma (Meeker et al. 2012; Kunis et al. 2013; Shechter et al. 2013; Ghersi-Egea et al. 2018; Thompson et al. 2022). However, evidence from Llovera et al. (2017) challenges this route: In a murine stroke model, the majority of periinfarct lymphocytes were shown to originate from the lateral ventricle or the choroid plexus of the lateral ventricle. When ventricular migration was inhibited by intraventricular matrigel injection, the numbers of periinfarct lymphocytes were not reduced. Consequently, an alternative pathway was proposed, suggesting that immune cells might migrate from choroidal blood vessels into the choroid plexus stroma and directly into the CNS parenchyma via its attachment regions. This is consistent with the description of a potential “functional leak” at the base of the choroid plexus based on ultrastructural observations by Brightman and Reese (1969).

Given that the attachment region of the choroid plexus might represent a potential conduit between the CNS parenchyma and the choroid plexus stroma, the first step to investigate this area is a thorough description of its morphology to evaluate whether this area contains a structure that could be relevant for the modulation of immune cell migration diffusion into the CNS environment.

In the CNS, barriers are commonly formed by astrocytes, known as the glia limitans. This boundary layer, composed of astrocyte endfeet and a basal lamina, exists as the glia limitans superficialis on the brain surface and as the glia limitans perivascularis along blood vessels (Sofroniew 2015). The astrocytes involved in the formation of the glia limitans superficialis have only recently been described as a highly specialised astrocyte subtype with distinctive gene expression profiles, potentially playing an essential role in brain development and transduction of inflammatory signals from the periphery (Hasel et al. 2025).

In this study, we have examined if glial structures are present at the attachment regions of the choroid plexus. Building on our previous work mapping these attachment regions in the murine CNS (Greiner et al. 2022), including three-dimensional reconstructions of the ventricular system and detailed localization of the attachment regions, we focused on the choroid plexus of the murine third and fourth ventricles, which exhibit a broader base and attachment region towards the brain parenchyma than the choroid plexus of the lateral ventricle. We conducted a morphological analysis of these areas in both murine and human tissues. Additionally, we assessed immune cell accumulation in these regions using a neuroinflammatory mouse model, aiming to shed light on the potential role of these attachment regions in immune cell migration.

## Materials and Methods

### Experimental Animals and Neuroinflammatory Mouse Model

For investigation of healthy tissue, female 10-week-old C57BL6/J mice (*n* = 5 for histological, *n* = 3 for ultrastructural studies and *n* = 5 for gene expression analysis) were obtained from the stockbreeding of the central animal facility of Rostock University Medical Center. The mice were maintained at a maximum of five animals per cage with food and water ad libitum. The mice were kept under standard conditions (12 h light/dark cycle, controlled temperature 22 °C ± 2 °C). Cages were changed once per week. All experimental procedures concerning animals were performed according to the Federation of European Laboratory Animal Science Associations recommendations, in accordance with the German Animal Welfare Law, and approved by the local authorities.

For investigation under neuroinflammatory conditions, C57BL6/J mice were obtained from Janvier Labs (Le Genest-Saint-Isle, France) and housed as described above. Female 10-week-old (19–23 g) mice were randomly assigned to the following groups: (i) control animals received a diet of standard rodent chow for the duration of the 7-week study (*n* = 5 for detailed histological assessment and *n* = 4 for gene expression analysis); (ii) Cuprizone/experimental autoimmune encephalomyelitis (CupEAE) animals (*n* = 5 for detailed histological assessment, *n* = 3 for ultrastructural studies and *n* = 6 for gene expression analysis) were fed a cuprizone diet (0.25% cuprizone [bis(cyclohexanone)oxaldihydrazone] Alfa Aesar, Thermo Fisher Scientific Inc.) mixed into ground standard rodent chow) for the first three weeks and then EAE was induced at the beginning of week five (Scheld et al. 2016). (iii) The preliminary study (Supplementary Fig. 3D) was performed in tissue of CupEAE mice (*n* = 8) that were fed Cuprizone two days prior to EAE induction and were fed continuously until peak of disease. To induce EAE, mice were immunized by subcutaneous injection of an emulsion of MOG35-55 peptide dissolved in complete Freund’s adjuvant followed by intraperitoneal injections of pertussis toxin in PBS on the day of and the day after immunization (Cat# EK2110, Hooke Laboratories, USA). Disease severity was scored daily based on EAE-induced clinical symptoms (tail and limb weakness or paralysis) as described in previous work and animals were dissected at peak of disease (i.e. scores do not rise for two consecutive days or scores decline) (Rüther et al. 2017). The animal experiments were authorized in accordance with § 8 of the German Animal Welfare Act (reference number of the Landesamt für Landwirtschaft, Lebensmittelsicherheit und Fischerei Mecklenburg-Vorpommern 7221.3-1.3-001/19 and reference number of the Regierung Oberbayern 55.2-154.2-2532-73-15) and all applicable animal welfare guidelines were complied with.

### Transcardial Perfusion

Mice were then anesthetized with an overdose of ketamine (750 mg kg^−1^ i.p.) and xylazine (50 mg kg^−1^ i.p.) and transcardially perfused with 20 ml of ice-cold phosphate-buffered saline (PBS) followed by 50 ml of phosphate-buffered 3.7% formaldehyde solution (pH 7.4). For histological studies brains were dissected from the skull and post-fixated in buffered formaldehyde solution overnight. For ultrastructural studies instead of formaldehyde solution mice were perfused with a phosphate-buffered fixing agent containing 2% glutaraldehyde and 1% formaldehyde. After opening the cranial calvaria, the heads were postfixated in the fixation solution at 4 °C for 24 h to ensure optimal preservation of brain morphology. The following day, the brains were dissected from the skull and stored in fixation solution at 4 °C. For laser capture microdissection studies the mice were perfused with PBS only and the brains were then removed natively and transferred directly into liquid isopentane, shock-frozen at −60 °C and stored at −80 °C.

### Human Post Mortem Samples

Post mortem tissue was sampled from body donations of the Institute of Anatomy (Rostock University Medical Center). The procedure was approved by the local ethics committee (reference number A 2021-0006). Whole brains from 5 donations (Table 1) were fixated in 3.7% buffered formalin for two weeks at 4 °C. Subsequently, tissue blocks with the attachment regions of all ventricles were sampled and histologically processed.

**Table 1:** Body donations

	Sex	Age at death	Cause of death	Post mortem interval until tissue processing
1	Female	84	Urothelial carcinoma	< 24 h
2	Male	84	Gastrointestinal bleeding	< 36 h
3	Female	87	Cardiorespiratory failure	< 36 h
4	Female	85	Tumor-related toxic multiple organ failure	< 24 h
5	Male	88	Aspiration	< 36 h

### Histology and Immunofluorescence Labeling

All histological studies and immunolabelings were performed using paraffin-embedded 5 μm-thick coronal brain sections. Sections used for evaluation of the third ventricle attachment region were located directly caudal to the subfornical organ (between Bregma − 0.82 mm and − 0.94 mm in the Mouse Brain Atlas of Paxinos and Franklin). Sections used for evaluation of the fourth ventricle were located at the cranial attachment of the choroid plexus to the cerebellum (Bregma − 6.12 mm in the Mouse Brain Atlas of Paxinos and Franklin).

For quantitative analyses, two consecutive sections per animal were analyzed and results were averaged. For hematoxylin and eosin (HE) staining, sections were deparaffinized, rehydrated and then incubated in Mayer’s hemalum solution for 10 min. After dipping in hydrochloric acid ethanol, sections were washed in running tap water. The sections were then washed in distilled water and incubated in eosin solution. The staining intensity of eosin was differentiated in 70% ethanol. After dehydration, the sections were mounted with Depex^®^.

For immunohistochemistry, sections were deparaffinized and rehydrated. Antigens were unmasked by microwave heating to the boiling point in tris(hydroxymethyl)aminomethane/ethylenediamine tetraacetic acid (Tris/EDTA) buffer (pH 9.0) for 10 min. The sections were washed in PBS and then incubated for 1 h with 5% normal goat serum in PBS. After draining the blocking serum, the sections were incubated overnight at 4 °C with the primary antibodies diluted in blocking solution (anti-GFAP, Abcam Cat# ab68428, RRID: AB_1209224, 1:200; anti-CD3, Abcam Cat# ab11089, RRID: AB_2889189, 1:250; anti-Laminin, Abcam Cat# ab11575, RRID: AB_298179, 1:300; anti-Ly6G, BioLegend Cat# 127602, RRID: AB_1089180, 1:250; anti-IBA1, FUJIFILM Wako Pure Chemical Corporation Cat# 019–19741, RRID: AB_839504, 1:2500). Appropriate negative controls (omission of primary antibody) were performed in parallel. The slides were treated with 0.35% hydrogen peroxide (H_2_O_2_) in PBS for 30 min. After washing in PBS, the slides were incubated in biotinylated secondary antibody (goat anti-rabbit IgG, Vector Laboratories Cat# BA-1000, RRID: AB_2313606, 1:200; goat anti-rat IgG, Vector Laboratories Cat# BA9400, RRID: AB_2336202, 1:200) for 1 h at ambient temperature and then in peroxidase coupled avidin-biotin complexes for 1 h (ABC kit; Vector Laboratories, Peterborough, UK). The antigenic sites were detected by a reaction with 3,3’-diaminobenzidine (DAKO, Hamburg, Germany) and H2O2 yielding a brownish deposit.

For immunofluorescence labeling, sections were treated as described above for all steps prior to primary antibody application and were then incubated overnight with two primary antibodies diluted in blocking solution (anti-Laminin, Abcam Cat# ab11575, RRID: AB_298179, 1:300; anti-GFAP, Sigma Cat# SAB2500462-100UG, RRID: AB_10603437, 1:400, anti-CD31, Dianova Cat# DIA-310, RRID: AB_2631039, 1:20; anti- αSMA, Abcam Cat# ab21027, RRID: AB_1951138, 1:100; anti-GFAP, Abcam Cat# ab4674, RRID: AB_1209224, 1:4000; anti-AQP4, Santa Cruz Cat# sc20812rb, RRID: AB_2274338, 1:500). Appropriate negative controls (omission of primary antibodies and cross controls of primary antibody and mismatched secondary antibody) were performed in parallel. After washing in PBS, the sections were incubated with the appropriate secondary antibodies diluted 1:250 in blocking serum (Alexa Fluor^TM^ 488 donkey anti-mouse, Abcam Cat# ab150109, RRID: AB_2571721; Alexa Fluor^TM^ 488 donkey anti-rabbit, Abcam Cat# ab150061, RRID: AB_2571722; Alexa Fluor^TM^ 594 donkey anti-rat, Abcam Cat# ab150156, RRID: AB_2890252; Alexa Fluor^TM^ 594 donkey anti-goat, Abcam ab150132, RRID: AB_2810222; Alexa Fluor^TM^ 647 donkey anti-rabbit, Abcam Cat# ab150063; RRID: AB_2687541; Alexa Fluor^TM^ 488 donkey anti-chicken, Invitrogen Cat# A78948, RRID: AB_2921070) for 2 h at room temperature. The sections were then washed in PBS and mounted in 4’,6-diamidino-2-phenylindole (DAPI)-containing mounting medium (Fluoroshield^TM^ with DAPI) for the staining of cell nuclei. Antibody specificity was assessed based on manufacturer validation data, previously published validation studies, and the expected cell type– and region-specific expression patterns. Appropriate negative controls were included.

All stained and processed sections were documented using a brightfield/epifluorescence microscope (Leica DM6 B, Germany) equipped with ×10, ×20 and ×40 objectives, using the software Leica Application Suite X (version 3.7.0.20979, 2019, Germany). Fluorescence imaging was performed using Leica filter systems TXR ET (excitation BP 560/40, dichroic LP 585, emission BP 630/75), L5 (excitation BP 480/40, dichroic 505, emission BP 527/30) and DAPI ET (excitation BP 350/50, dichroic LP 400, emission BP 460/50). Exposure times and acquisition settings were kept constant within each experimental series to allow direct comparison between groups. All images shown are representative of multiple independent samples acquired under identical imaging conditions.

For cell density measurements, the choroid plexus vili were manually encircled to calculate the choroid plexus area. The choroid plexus base was defined as the part of the choroid plexus opposed to the attachment region that is not structured in choroid plexus vili and is characterized by more and larger blood vessels than the rest of the choroid plexus stroma.

**Fig. 1:**
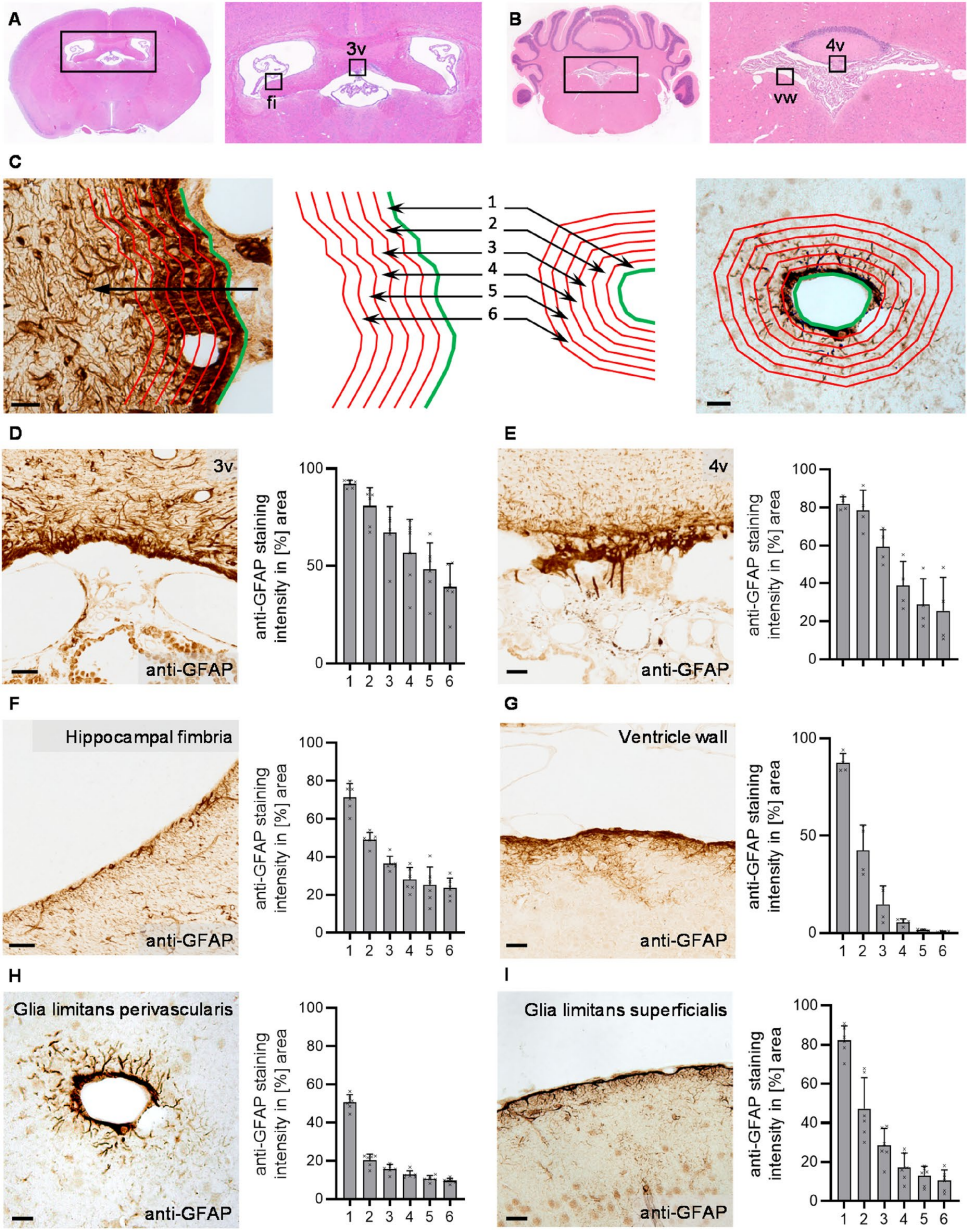
Astrocyte accumulation at the murine choroid plexus attachment region. **A** Localization of the attachment region of the choroid plexus of the third ventricle and the reference region in the hippocampal fimbria (fi); **B** localization of the attachment region of the choroid plexus of the fourth ventricle and its reference region in the ventricle wall (vw). **C** The optical density of anti-glial fibrillary acidic protein (GFAP) immunohistochemical labelling was evaluated in six zones of 25 µm width, exemplarily shown for the attachment region and the glia limitans perivascularis. **D**–**I** Exemplary image of anti-GFAP immunolabelling in control mice and graphical representation of the optical density analysis in zones 1–6; **D** attachment region of the choroid plexus in the third ventricle; **E** attachment region of the choroid plexus in the fourth ventricle; **F** fimbria hippocampi facing the lateral ventricle, used as a reference region for **D**; **G** wall of the fourth ventricle, used as a reference region for **E**; **H** perivascular glia limitans; **I** cortical superficial glia limitans. All analyses were done in untreated mice. Scale bars in **C**–**I** 50 μm. *n* = 4–5 mice

Optical density analyses were performed to evaluate anti-GFAP immunohistochemical labelling with ImageJ (version 1.53c, NIH, Bethesda, MD, USA). A polygonal line was drawn on the surface to be analyzed, which was then shifted 6 times in parallel by 25 μm using a directional arrow (Fig. 1C). This created 6 identical parallel regions of interest (ROI), allowing consistent sampling of the attachment region while preserving spatial resolution along the analyzed surface. To evaluate the optical density of anti-GFAP positive structures along vessels, a ring was drawn in the inner edge of the vessel, which was then extended 6 times in radius by 25 μm to create 6 ring-shaped ROIs with a width of 25 μm. To determine the optical density in the individual ROIs, images were converted into binary black and white formats using automated thresholding. The proportion of black pixels per area then corresponds to the optical density.

**Fig. 2:**
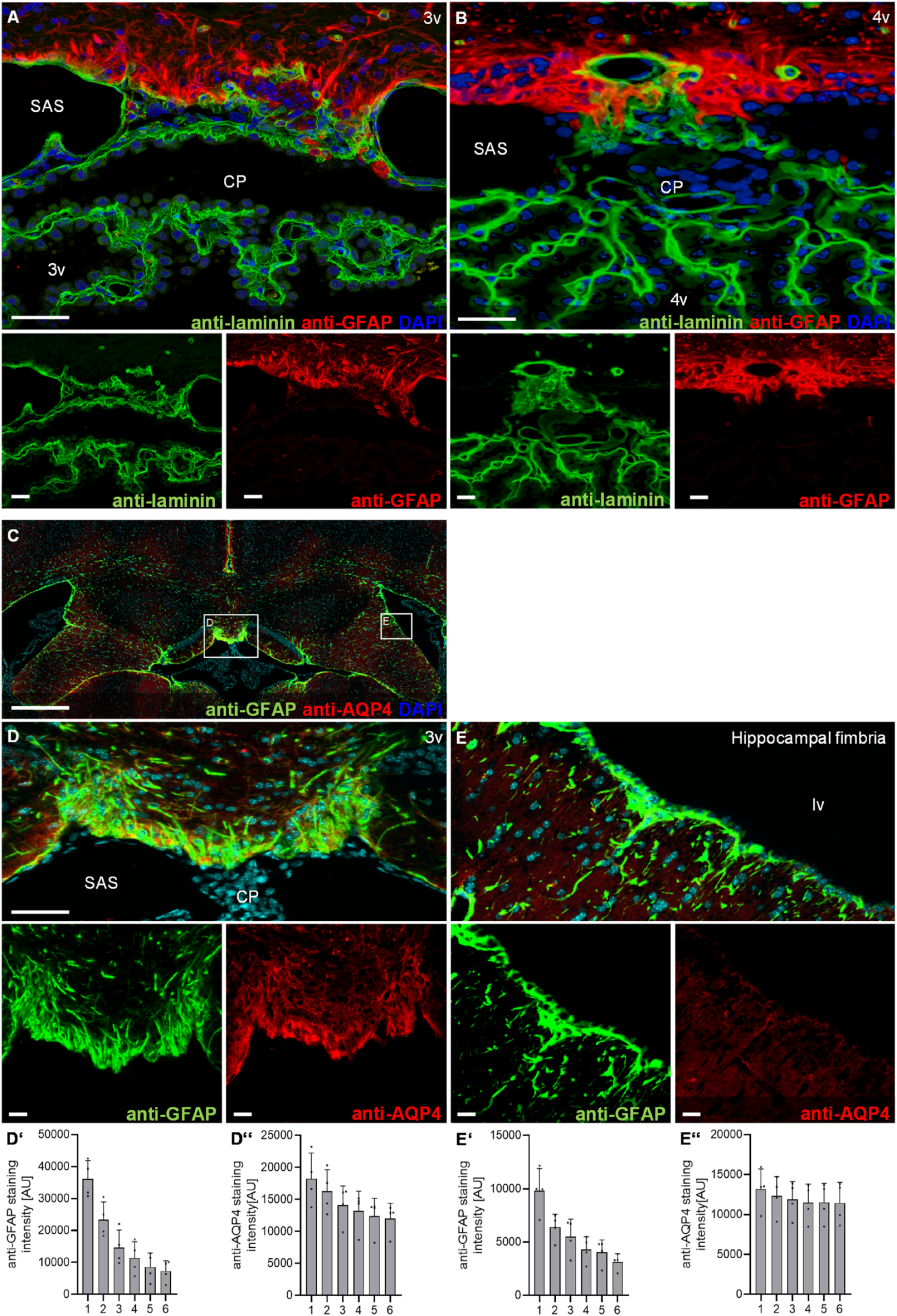
Basal lamina and aquaporin 4 at the murine choroid plexus attachment region. **A**, **B** Immunofluorescence double staining with anti-laminin (green) and anti-glial fibrillary acidic protein (GFAP, red) at the attachment region in the third ventricle **A** and fourth ventricle **B** in control mice. **C**–**E** Immunofluorescence double staining with anti-GFAP (green) and anti-aquaporin 4 (AQP4) at the attachment region in the third ventricle **D** and the hippocampal fimbria **E**. (**D’**–**E’’**) Fluorescence intensity profiles in 6 zones of 25 μm width were plotted to illustrate the spatial distribution of AQP4 and GFAP labeling relative to the attachment region. Abbreviations: 3v third ventricle, 4v fourth ventricle, CP choroid plexus, lv lateral ventricle, SAS subarachnoid space. Scale bars in **A**–**E** 50 μm. *n* = 4 mice

The fluorescence signal intensity of the anti-GFAP and anti-AQP4 immunofluorescence labelling (see Fig. 2D, E) was quantified using the software QuPath (version 0.5.1) (Bankhead et al. 2017). For this analysis, six parallel ROIs were defined as described above, and the mean fluorescence intensity of the anti-GFAP and anti-AQP4 signal was measured within each ROI.

Blinding of experimenters to the experimental groups was not feasible due to the nature of the experimental setup. Image quantification was therefore performed using automated and predefined analysis pipelines to minimize subjective bias.

### Electron Microscopy

For transmission electron microscopy (TEM), the tissue was roughly cut to the region to be analyzed before embedding. The brains were removed from the fixative and were either cut into thin slices with a razor blade using a mouse brain mould or were alternatively embedded in hot agarose (2.5%) for vibratome sectioning. After complete hardening of the agarose blocks, 200 μm thick slices were prepared on a vibratome (Leica VT1000 S, Germany) as series of consecutive sections and stored in fixative. Each brain slice was preliminarily washed several times in a phosphate buffer (pH = 7.3) and stored overnight in the buffer. The blocks were then post-fixated in a 1% osmium tetroxide solution (Carl Roth, Germany) for 2 h and after washing in distilled water dehydrated in an ascending series of acetone. The osmium tetroxide solution was also used to contrast the tissue samples, as the contrast in transmission electron imaging depends on the atomic number, i.e. on the incorporation of various heavy metals into the respective sample structure. The tissue was then incubated in an acetone/epon mixture (48% Epon 812, 30% methylnadic anhydride, 20.7% 2-dodecenylsuccinic acid anhydride, 1.3% 2,4,6-tris(dimethylaminomethyl)phenol; all components from Serva, Heidelberg, Germany) for infiltration until the acetone had completely evaporated. The prepared brain slices were embedded in the synthetic resin Epon, which serves as a hard embedding medium. Further processing included the trimming of the resin blocks (Leica EM Trim2, Leica Microsystems, Wetzlar, Germany) and subsequent sectioning on an ultramicrotome (Ultracut S, Reichert, Wien, Austria) using a diamond knife (Diatome, Nidau, Switzerland). To visualize and select the areas for ultrastructural examination, semithin sections with a thickness of 0.5 μm were stained with toluidine blue. Ultrathin sections of about 50–70 nm thickness were transferred to copper grids and stained with uranyl acetate and lead citrate before being examined with a Zeiss EM902 transmission electron microscope operated at 80 kV (Carl Zeiss, Oberkochen, Germany). Digital images were acquired with a side-mounted 1 × 2k FT-CCD Camera (Proscan, Scheuring, Germany) using iTEM camera control and imaging software (iTEM version number 1187, Olympus Soft Imaging Solutions, Münster, Germany).

### Statistical Analysis

All data are given as arithmetic means ± standard deviation. Statistical testing was performed using Prism 8.0.2 (GraphPad Software Inc., San Diego, CA, USA) with confidence intervals of 0.05. Sample sizes (n) refer to biological replicates unless otherwise stated and are reported in the Methods section and in the corresponding figure legends. No a priori statistical power calculation was performed; sample sizes were determined based on prior experience with similar experimental paradigms and effect sizes reported in the literature. The Shapiro-Wilk test was applied to test for normality distribution. Homogeneity of variances was evaluated using the Brown–Forsythe test. Ordinary one-way ANOVA with Tukey’s multiple comparisons test was applied for normally distributed data. If standard deviations were significantly different, Brown-Forsythe and Welch ANOVA test with Dunnett’s T3 multiple comparisons test was applied. Kruskal-Wallis test with Dunn’s multiple comparisons test was applied for not normally distributed data. P-values of ≤ 0.05 were considered to be statistically significant. The following symbols indicate the level of significance: *p ≤ 0.05, **p ≤ 0.01, ***p ≤ 0.001. No outliers were excluded from the analyses.

## Results

### Morphological Examination of the Choroid Plexus Attachment Region

In a first step, we used immunolabelling to investigate the density of glial fibrillary acidic protein (GFAP)-positive astrocytes at the attachment regions of the choroid plexus in the third (Fig. 1A, Supplementary Fig. 1 A) and fourth ventricle (Fig. 1B, Supplementary Fig. 1B) in the healthy mouse brain. In this manuscript, the term “attachment region” refers to the area of brain parenchyma that is directly adjacent to the choroid plexus stroma. As reference regions, we selected areas of the ventricular walls (hippocampal fimbria for the third ventricle and the ventricular wall of the hindbrain for the fourth ventricle) and well-established glial barrier structures (perivascular glia limitans of parenchymal blood vessels and superficial glia limitans of the cortical surface). GFAP immunoreactivity was analyzed across six regions of interest (ROIs) extending from the surface towards the parenchyma (Fig. 1C) to characterize the spread of astrocytic structures into the parenchyma.

In all analyzed regions, a high optical density of GFAP-positive cells was detected in the first ROI, which represents the outer lining of the respective border structure. The subsequent decline in optical densities across the following ROIs indicates the extent of GFAP-immunoreactive zones into the parenchyma. The attachment regions in both the third and fourth ventricles displayed a broader area of high GFAP optical density compared to the reference regions. At the attachment region of the third ventricle (Fig. 1D), the optical density in the first ROI was 92.1% on average, with the optical density only falling below half of this value in the 6th ROI. At the attachment region of the fourth ventricle (Fig. 1E), the optical density in the first ROI was 82.0%; the optical density did not fall below half of this value until ROI 4. In the selected reference regions at the ventricular walls (Fig. 1F, G), the optical density was 72.5 and 87.3% and fell to half of these values in the third ROI at the latest. In contrast, the glia limitans perivascularis (Fig. 1H) had an innermost ROI optical density of 50.6%, while the glia limitans superficialis (Fig. 1I) showed 82.2%, both falling below half of these values by the second ROI. Thus, the choroid plexus attachment regions in the third and fourth ventricles of the murine brain exhibit a zone of GFAP-immunoreactivity that extends further into the parenchyma than in any of the reference regions.

Glial barriers of the CNS are composed of astrocytes and a basal lamina. In a second step, we therefore investigated if the detected accumulation of GFAP-positive cells is in any spatial relationship to a basal lamina. To this end, we performed immunofluorescence double staining with anti-GFAP and anti-laminin at the choroid plexus attachment regions in the third and fourth ventricle. A continuous laminin layer was observed along the GFAP-positive cell accumulation in both ventricles (Fig. 2A, B). Astrocytic processes of the superficial and perivascular glia limitans are characterized by aquaporin 4 (AQP4) expression. We therefore performed immunofluorescence double staining of anti-GFAP and anti-AQP4 at the choroid plexus attachment regions of the third ventricle, using the hippocampal fimbria as a reference region of the ventricular wall. Strong anti-AQP4 labelling was detected at the attachment region aligned with the GFAP-positive structures, whereas anti-AQP4 labelling along the ventricular wall was comparatively faint (Fig. 2C–E). Fluorescence intensity profiles were plotted to illustrate the spatial distribution of AQP4 labeling extending from the attachment region into the adjacent parenchyma (Fig. 2D’, D’’, E’, E’’).

### Ultrastructure of the Choroid Plexus Attachment Region

**Fig. 3:**
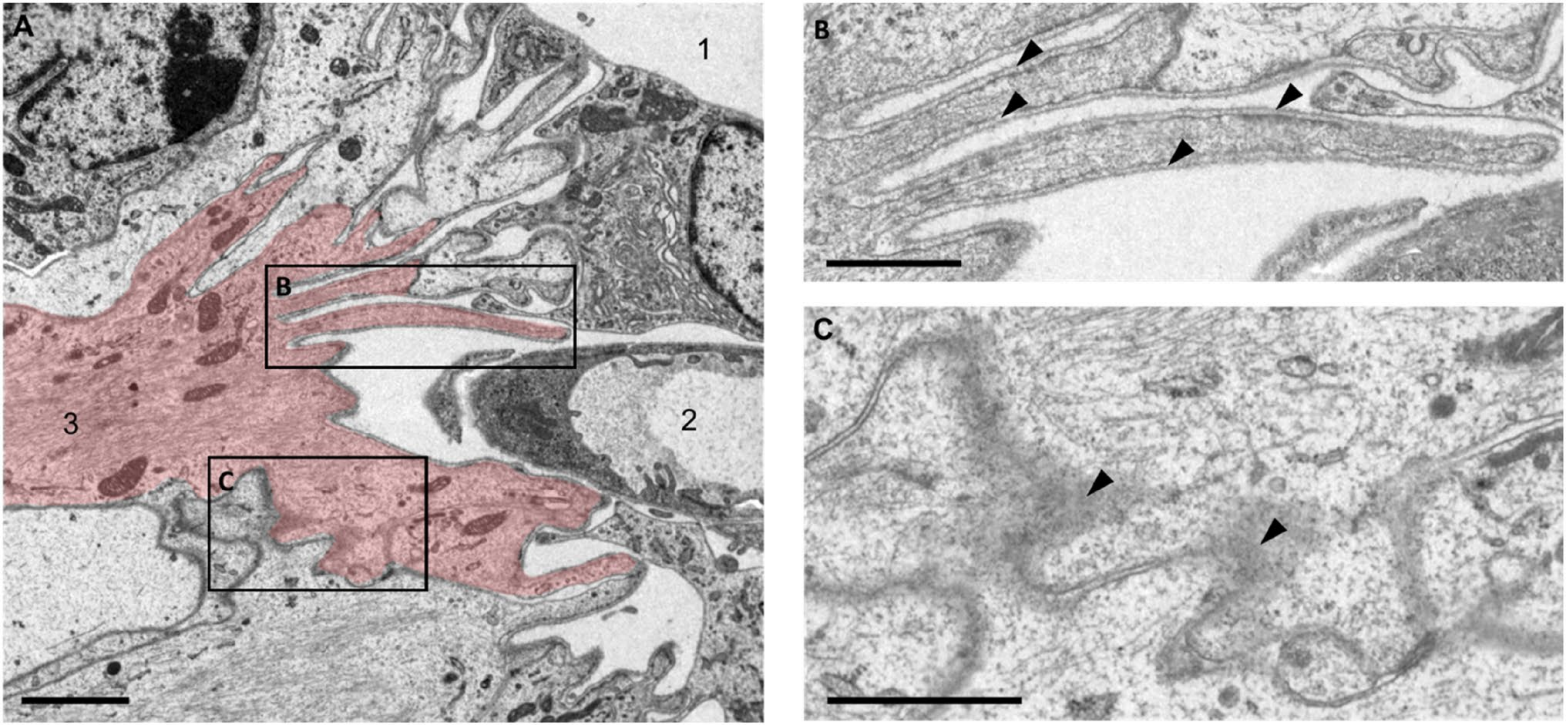
Ultrastructural morphology of the murine choroid plexus attachment region. **A** Exemplary electron micrograph of the attachment region in the third ventricle of a control mouse. 1: third ventricle lumen, 2: choroidal vessel, 3: astrocyte (red) with intermediary filaments and mitochondria. **B** astrocytic processes with a continuous basal lamina (arrowheads) **C** cell membrane of the astrocyte with dense cell-cell contacts to the neighboring cell (black arrowheads). Scale bars in **A** 2 μm, in **B**, **C** 1 μm. *n* = 3 mice

To further characterize the morphology of the choroid plexus attachment region, we obtained transmission electron microscopy (TEM) images of the third ventricle attachment region. Complex, cross-linked cells, likely astrocytes based on their high content of intermediary filaments, were identified within the attachment region (Fig. 3A, red). Mitochondria were visible in these cells, and structures consistent with junctional complexes were observed interconnecting processes of neighboring astrocytes (Fig. 3C, arrowheads). These astrocytes were surrounded by a continuous basal lamina that extended along multiple astrocytes and their processes (Fig. 3B, arrowheads). Thus, ultrastructural analysis confirmed the presence of a continuous basal lamina and demonstrated close cell-cell contacts among astrocytes at the attachment region. Additionally, tight junction components (Jam1, Cldn1), gap junctions (Gja1) and basal lamina components (Lamb1) were detected within the choroid plexus attachment region on the gene expression level (Supplementary Fig. 2). As these transcriptomic data were generated using amplification-based approaches, they should be interpreted as supportive rather than quantitative evidence.

### Immune Cell Accumulation at the Choroid Plexus Attachment Region

It is currently unclear if the choroid plexus attachment regions are relevant for immune cell migration into the CNS parenchyma. We therefore aimed to examine if this region is of potential relevance for immune cell accumulation in a neuroinflammatory mouse model. We utilized the CupEAE neuroinflammatory mouse model, which induces peripheral immune cell recruitment to various regions of the murine forebrain. The morphology and gene expression at the attachment region were not significantly altered in the CupEAE model (Supplementary Fig. 3A–C). While the model is associated with generalized astroglial activation, neither overt morphological changes nor increased GFAP transcript levels were observed at the attachment region. GFAP immunoreactivity does not necessarily correlate with GFAP transcript abundance, as astrocytic reactivity involves cytoskeletal remodeling and hypertrophy of GFAP-positive processes (Sofroniew and Vinters 2010; Hol and Pekny 2015), and mRNA levels do not always predict protein abundance (Liu et al. 2016).

In a first preliminary experiment, we investigated if immune cells accumulate in brain regions close to choroid plexus attachment regions. We observed higher densities of CD3-positive cells in periventricular regions near the choroid plexus attachment regions (e.g., the hippocampal formation and fimbria) compared to regions without spatial relation to the choroid plexus (e.g., periventricular caudoputamen complex and habenulae) (Supplementary Fig. 3D).

**Fig. 4:**
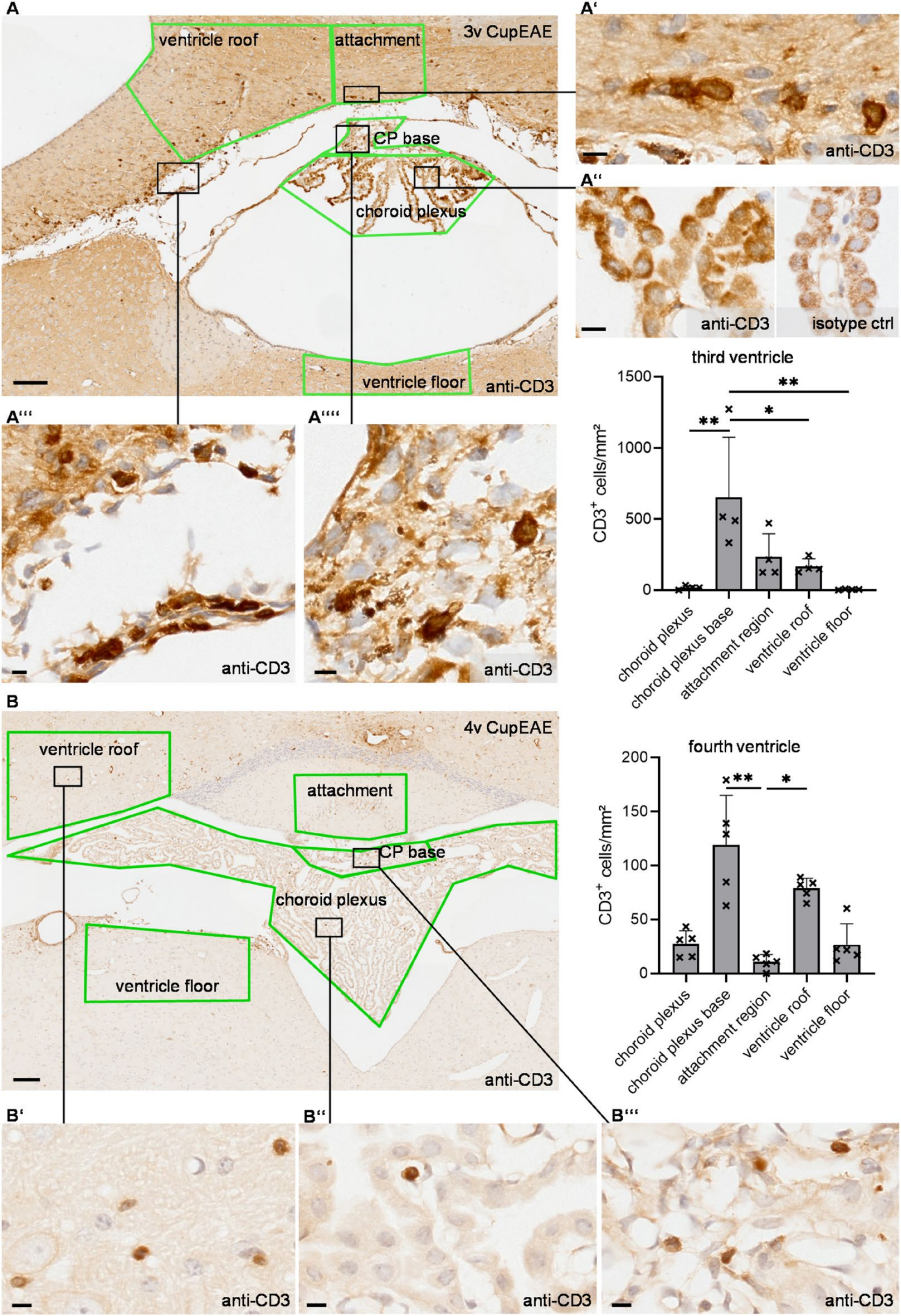
Immune cell accumulation at the choroid plexus attachment region in a neuroinflammatory mouse model. **A**, **B** Representative anti-CD3 immunolabelling with hematoxylin counterstaining in cuprizone/experimental autoimmune encephalomyelitis (CupEAE) mice of the attachment region in the third ventricle **A** and fourth ventricle **B**. Magnifications show the membranous staining pattern on CD3-positive cells (T cells) in the attachment region **A’**, the subarachnoid space **A’’’**, the choroid plexus (CP) base **A’’’’** and slight unspecific labelling of choroid plexus epithelial cells both in anti-CD3 labelling and isotype control **A’’**. All regions for cell density quantification are labelled in green. Diagrams show the density of CD3-positive cells per square millimeter in the third and fourth ventricle. **A** Ordinary one-way ANOVA followed by Tukey’s multiple comparisons test (F(4, 15) = 6.71, *p* = 0.0026) **B** Kruskal–Wallis test followed by Dunn’s multiple comparisons test (H(4) = 19.86, *p* = 0.0005) Scale bars in **A**, **B** 100 μm, *n* = 5 mice

Based on these findings, we closely examined immune cell distribution at the third and fourth ventricle attachment regions compared to adjacent reference regions. In control animals, CD3-positive cells were mostly absent in the brain parenchyma of the analyzed regions. In the CupEAE model, CD3-positive cell densities were generally higher in the attachment region of the third ventricle than that of the fourth ventricle (Fig. 4A, B). At the third ventricle attachment region, the average density of CD3-positive cells was 234.5 ± 163.3 cells/mm^2^, compared to 167.9 ± 52.6 cells/mm^2^ in the corresponding reference region at the ventricle roof and 4.6 ± 3.7 cells/mm^2^ in the reference region at the ventricle floor. Within the choroid plexus of the third ventricle, CD3-positive cell density was 17.3 ± 13.9 cells/mm^2^, however, the choroid plexus base, i.e. the choroid plexus stroma directly opposed to the attachment region harbored 653.1 ± 421.2 cells/mm^2^ CD3-positive cells/mm². Of note, the choroid plexus base is a small region, therefore low total cell numbers lead to considerable variability in cell density.

In the fourth ventricle attachment region, the average cell density of CD3-positive cells was 10.7 ± 6.9 cells/mm^2^, compared to 78.9 ± 9.4 cells/mm^2^ in the reference region at the ventricle roof and 26.8 ± 19.3 cells/mm^2^ CD3-positive cells/mm^2^ at the ventricle floor. CD3-positive cell density within the choroid plexus was 17.2 ± 8.9 cells/mm^2^ with 99.8 ± 56.7 cells/mm^2^ CD3-positive cells/mm² at the choroid plexus base. Of note, high densities of parenchymal CD3-positive cells did not coincide with high blood vessel densities (Supplementary Fig. 5).

Of note, the stroma of the choroid plexus is contiguous to the subarachnoid space. In the murine brain, the subarachnoid compartment forms infoldings that extend into deeper regions between the telencephalic cortex and diencephalic/midbrain structures (Greiner et al. 2022; Pleskac and Engelhardt 2026). Regions of subarachnoid space lateral to the attachment region showed high numbers of CD3-positive immune cells (Fig. 4A’’’). Unfortunately, the area of subarachnoid space was heavily affected by artifacts causing enlargement and disruption of this space to such an extent that it is not possible to define a reliable region for quantifying cell densities. Therefore, quantitative analyses are restricted to the choroid plexus and brain parenchyma.

**Fig. 5:**
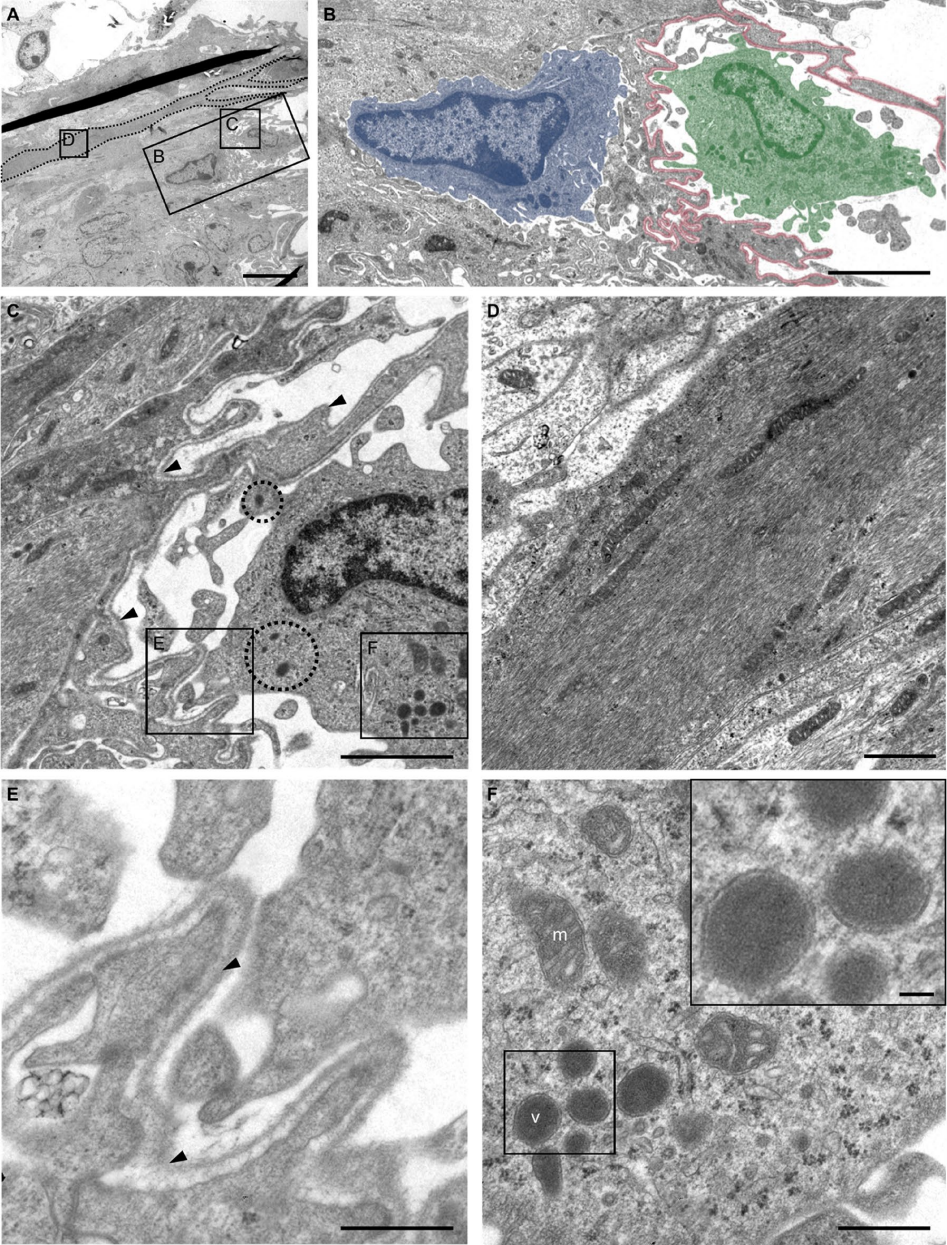
Electron microscopic examination of the attachment region in the third ventricle in the murine CupEAE model. **A** Electron micrograph of the attachment region in the third ventricle in a cuprizone/experimental autoimmune encephalomyelitis (CupEAE) mouse. Brain parenchyma is located at the left side, the choroid plexus/third ventricle is located on the right side. A cellular process densely packed with intermediary filaments which most likely belongs to an astrocyte is marked by a dotted outline. The location within the attachment region is shown in Supplementary material 3E. **B** Magnification of **A** as indicated. Phagocyte-like cells are located on the parenchymal side of the basal lamina (blue overlay) and on the choroid plexus side of the basal lamina (green overlay). The basal lamina is marked in red. Magnification in **C** shows the continuous basal lamina between the phagocytes (arrowheads) and vesicles of high electron density (circles) within the choroidal phagocyte. **D** Magnification of the astrocyte process with densely packed intermediary filaments and mitochondria. **E** Magnification of the basal lamina between the two phagocytes as indicated in **C**. **F** Magnification of vesicles (v) and mitochondria (m) inside the choroidal phagocyte as indicated in **C**. Scale bars in **A** 10 μm, **B** 5 μm, **C** 2 μm, **D** 1 μm, **E**, **F** 500 nm, 100 nm in inset. *n* = 3 mice

To gain more insight into the localization of peripheral immune cells in relation to the astrocytic structure in the attachment region, we performed ultrastructural analysis of the attachment region of the murine third ventricle in the neuroinflammatory CupEAE model (Fig. 5A, Supplementary Fig. 3E). The morphology of the attachment region appeared similar to the physiological state, with astrocytes containing abundant intermediary filaments (exemplary astrocytic process marked by dotted outline in Fig. 5A). The intermediary filaments appear more densely packed in many astrocytes (Fig. 5D) compared to control conditions which can be explained by a generalized astrogliosis in the CupEAE model. Comparable to the physiological state, astrocytes and their processes were surrounded by a continuous basal lamina (Fig. 5C, arrowheads and Fig. 5E). Cells with phagocyte-like morphology (Fig. 5B, blue and green overlays) were identified in the transition zone between the choroid plexus stroma and CNS parenchyma. Their ultrastructural appearance is consistent with either granulocytes or macrophages. Immunolabelling for Ly6G and Iba1 confirmed the presence of both granulocytes and macrophages at the attachment region (Supplementary Fig. 4). Ly6G-positive cells were observed at the attachment site and within the adjacent subarachnoid space, whereas the parenchyma was largely devoid of Ly6G-positive cells. In contrast, IBA1-positive cells were abundant in the parenchyma, representing activated microglia and presumably also infiltrating peripheral monocytes/macrophages, and were additionally present at the choroid plexus attachment region and in the subarachnoid space. As the ultrastructural features do not allow a definitive distinction between granulocytes and macrophages, the cells observed in the electron micrographs are hereafter referred to as phagocytes.

Ultrastructural analysis further revealed phagocyte-like cells on both sides of the basal lamina (red overlay in Fig. 5B) with noticeable spatial proximity to the basal lamina which is indicative for direct interaction with this structure. In Fig. 5B, the right phagocyte (green overlay) is located on the choroidal side of the basal lamina, while the left one (blue overlay) resides on the parenchymal side of the basal lamina. Of note, the choroidal phagocyte displayed densely packed cytoplasmic vesicles oriented towards the basal lamina (Fig. 5F).

### Morphology of the Choroid Plexus Attachment Region in the Human Brain

**Fig. 6:**
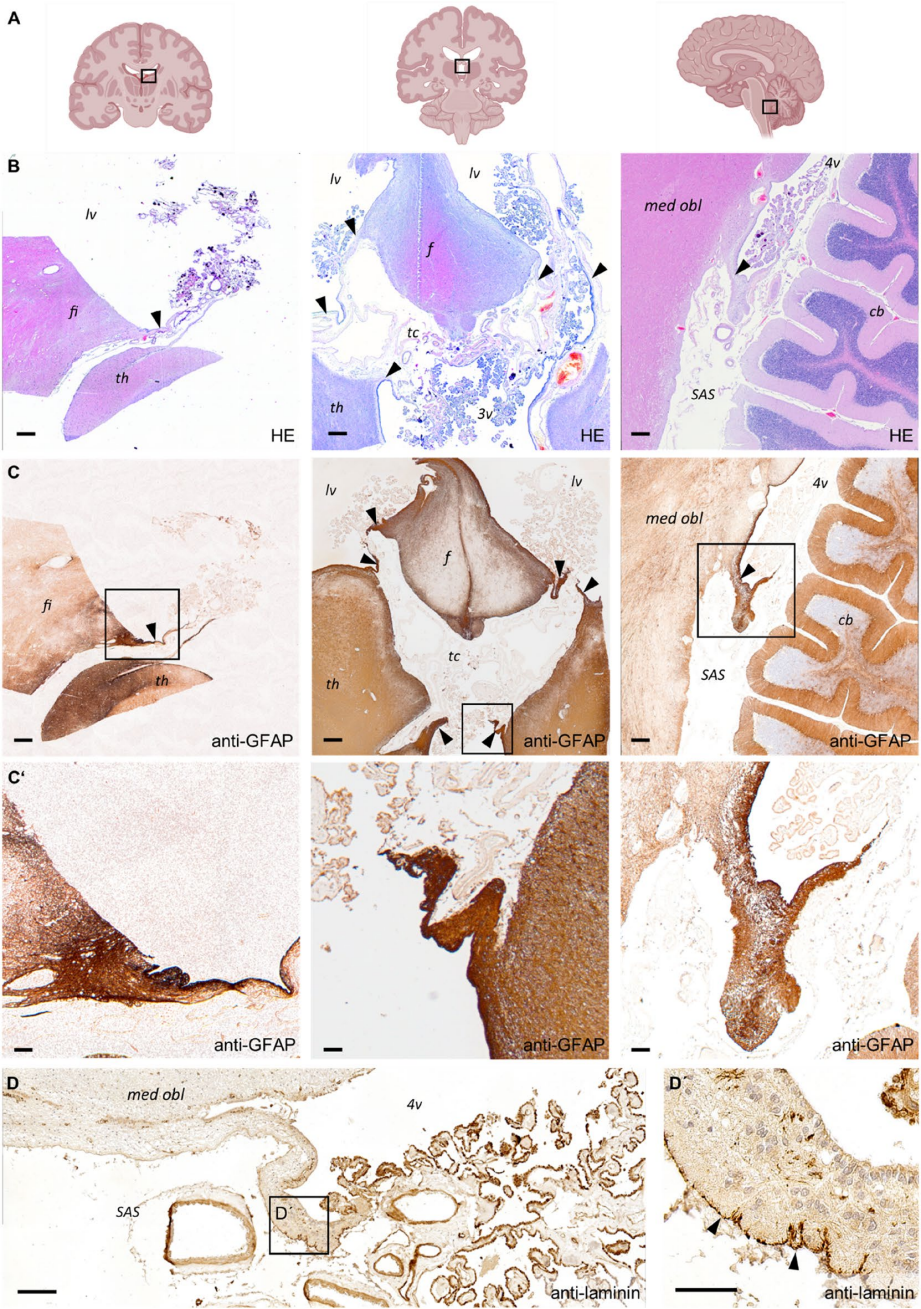
The attachment region of the choroid plexus in the human brain. **A** schematic representation of the sectional planes for lateral ventricle, third ventricle and fourth ventricle. **B** Hematoxylin and eosin (HE) overview staining. Tissue bridges of choroid plexus attachment are marked with arrowheads in all images. **C** Anti-glial fibrillary acidic protein (GFAP) immunolabelling for visualization of astrocytes, **C’** with magnification as indicated in **C**. **D** Representative anti-laminin immunolabelling of the attachment region of the fourth ventricle with magnification (**D’**) as indicated in **D**. Basal lamina directed towards the subarachnoid space is marked with arrowheads. Abbreviations: 3v third ventricle, 4v fourth ventricle, cb cerebellum, f fornix, fi fimbria hippocampi, lv lateral ventricle, med obl Medulla oblongata, th thalamus, SAS subarachnoid space, tc tela choroidea. Scale bars in **B**, **C** 100 μm, in **C’** 50 μm, in **D** 200 μm and in **D’** 50 μm. Created with BioRender.com

After demonstration of a border structure comprising astrocyte accumulation and a continuous basal lamina in the murine choroid plexus attachment region, we examined the morphology of the attachment regions of the human choroid plexus (Fig. 6A). In the human CNS, the choroid plexus is attached to tissue bridges named Taeniae (Nagata et al. 1988; Wen et al. 1998) (Fig. 6B, arrowheads). The human choroid plexus of the lateral ventricles is attached to the thalamus via the Taenia choroidea and to the fornix via the Taenia fornicis or, in its temporal aspect, to the hippocampal fimbria via the Taenia fimbriae which is continuous to the Taenia fornicis. The stroma of the lateral ventricle choroid plexus is continuous to the stroma of the third ventricle. At the roof of the third ventricle, this stromal tissue is called Tela choroidea and contains numerous blood vessels (Fig. 6B, central image, tc). Both the choroid plexus stroma of the lateral ventricle and the choroid plexus stroma of the third ventricle are continuous to the Tela choroidea. The choroid plexus of the fourth ventricle is attached to the medulla oblongata by structures comparable to the Taeniae of the lateral ventricle (Fig. 6B, arrowheads in right image). We focused our analysis on the two components of glial structures that we found in the murine situation: astrocytic accumulation and an associated basal lamina.

Immunohistochemical labelling of GFAP in tissue samples from body donations revealed high immunoreactivity against GFAP in all choroid plexus attachments/Taeniae (Fig. 6C), indicating a dense accumulation of astrocytes. Immunohistochemical analysis of laminin revealed a basal lamina localized different than in the murine situation. Laminin immunoreactivity was detected at the base of choroid plexus epithelial cells and around choroidal blood vessels as expected (Fig. 6D). However, in human tissue, laminin immunoreactivity did not form a barrier between the choroid plexus and the astrocytic attachment region, but was instead detected on the outer surface of the Taeniae connecting to the choroid plexus. Laminin immunoreactivity was only detected on surfaces facing the subarachnoid space, not at the ventricular surface. This finding is consistent with the presence of a basal lamina in the superficial glia limitans covering the brain surface directed toward the subarachnoid space. In contrast, the ventricular walls, which are covered with ependyma, lack a basal lamina.

## Discussion

We observed a glial accumulation at the attachment region of the choroid plexus. This region was characterized by GFAP and AQP4 expression, and by the presence of a continuous basal lamina demonstrated both by laminin immunoreactivity and ultrastructural analysis. In addition, gene expression and ultrastructural examination indicated the presence of junction-related components and intercellular junctional complexes.

The astrocytic accumulation at the choroid plexus attachment region aligns with the general morphology of the glia limitans superficialis and glia limitans perivascularis. However, it extends considerably farther into the parenchyma and occupies a broader zone than these established glial boundaries. It therefore remains unclear whether this glial accumulation represents part of the glia limitans superficialis or a region-specific specialization.

The glia limitans superficialis lines the CNS surface facing the subarachnoid space. Notably, the subarachnoid space folds into the depth of the CNS around the third ventricle, establishing continuity between the leptomeninges and the choroid plexus stroma. In this anatomical context, the astrocytic accumulation described here may reflect a continuation of the glia limitans superficialis. More detailed morphological studies will be required to determine whether this region represents an extension of the glia limitans superficialis or a distinct regional specialization.

This study did not assess the functional properties of the astrocytic accumulation in regard to barrier properties between the choroid plexus stroma and brain parenchyma. In the CupEAE model, CD3-positive T cells showed a tendency to accumulate in brain regions adjacent to choroid plexus attachment regions, and particularly high densities were found within the choroid plexus stroma directly opposite the attachment region. Ultrastructural analysis further revealed phagocyte-like cells located on both sides of the continuous basal lamina. Stromal phagocytes displayed a polarized arrangement of cytoplasmic vesicles oriented toward the basal lamina and were positioned in close apposition to it. The relevance of this observation for potential interactions between immune cells and the basal lamina will require dedicated functional studies.

While this immune cell distribution pattern highlights the spatial proximity of immune cells to the attachment region, it does not allow conclusions regarding their direction of movement or potential migration routes. Notably, the CupEAE model is known to exhibit heterogeneous effects with respect to immune cell invasion across different forebrain regions, which may obscure detectable migration patterns (Scheld et al. 2016). Accordingly, the functional role of the observed glial structure with respect to barrier properties or immune cell migration remains speculative and cannot be resolved based on the present data. Further studies employing tracer distribution analyses and cell-labeling approaches will be required to define barrier properties and thereby clarify the relevance of this region for immune cell migration under neuroinflammatory conditions.

In human brains, the attachment region of the choroid plexus also showed high GFAP expression; however, the organization of the basal lamina differs substantially from the murine situation. It therefore remains uncertain whether the astrocytic accumulation with basal lamina association at the choroid plexus base represents a murine-specific feature, which could contribute to interspecies differences in immune cell migration during neuroinflammation.

The role of the choroid plexus in human neurological disease has received increasing attention in recent years, particularly regarding alterations in CSF production and the glymphatic system (Carlstrom et al. 2022; Liu et al. 2022). Inflammatory changes of the choroid plexus have likewise been reported across several disorders. For example, disease-associated variation in choroid plexus gene expression, including inflammation-related genes, has been observed in post-mortem tissue from patients with Alzheimer’s disease, Huntington’s disease, and frontotemporal dementia (Stopa et al. 2018). In multiple sclerosis, T cells and granulocytes accumulate within the choroid plexus (Rodríguez-Lorenzo et al. 2020), and MRI-detectable choroid plexus swelling correlates with lesion load and may predict disease progression (Fleischer et al. 2021; Müller et al. 2022; Luessi et al. 2024). Given this involvement across diverse, particularly neuroinflammatory, conditions, the compartmentalization of the choroid plexus stroma and its role in immune cell migration demands further investigation in future studies.

This study provides a detailed anatomical characterization of the attachment region of the choroid plexus, with a particular focus on astrocytic accumulation and basal lamina organization. This transition zone between the choroid plexus stroma and the brain parenchyma is of considerable interest, as it may represent an entry route for immune cells into the CNS. The recent identification of a specialized subpopulation of choroidal fibroblasts at the base of the choroid plexus (Weller et al. 2018; Verhaege et al. 2026) further highlights the potential complexity of this region. Future studies will be required to assess whether this area constitutes a vulnerable site for immune cell entry during neuroinflammatory conditions.

## Supplementary Information

Below is the link to the electronic supplementary material.

Supplementary Material 1

## Data Availability

The raw data supporting the conclusions of this article will be made available by the authors, without undue reservation.

## Abbreviations

3v: Third ventricle
4v: Fourth ventricle
Cb: Cerebellum
CNS: Central nervous system
CP: Choroid plexus
CSF: Cerebrospinal fluid
EAE: Experimental autoimmune encephalomyelitis
F: Fornix
fi: Hippocampal fimbria
hf: Hippocampal formation
i.p.: Intraperitoneal
lv: Lateral ventricle
med: Obl medulla oblongata
PBS: Phosphate-buffered saline
PCR: Polymerase chain reaction
ROI: Region of interest
SAS: Subarachnoid space
tc: Tela choroidea
TEM: Transmission electron microscopy
th: Thalamus
vw: Ventricle wall
