# Supplementary material for: Astrocytic Accumulation at the Choroid Plexus Attachment Region
Source: Cell Mol Neurobiol. 2026 May 18;46:88. doi: 10.1007/s10571-026-01742-6 (PMC13184079; doi:10.1007/s10571-026-01742-6)
Supplement: Supplementary file 1 — Supplementary Material 1 [file 10571_2026_1742_MOESM1_ESM.pdf]

Supplementary Material

Supplementary Figure 1

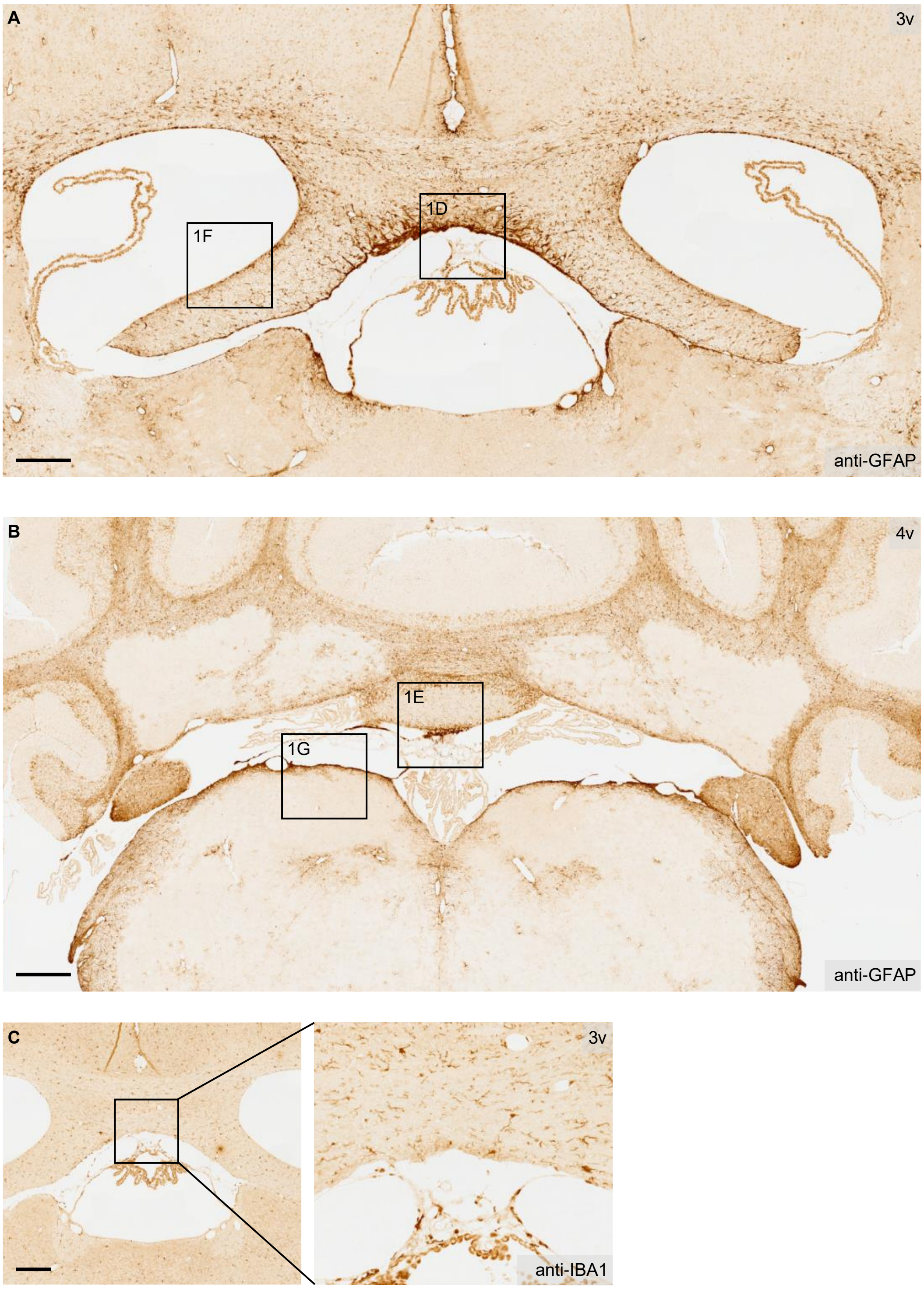

Supplementary Figure 1: Astrocytic and microglial cell accumulation at the attachment reagions of the choroid plexus

(A) and (B) Representative anti-GFAP immunohistochemical labellings of the third ventricle (A) and the fourth ventricle (B) in a control animal. Magnifications shown in Figure 1 are marked by squares. (C) Representative anti-IBA1 immunohistochemical labelling of the choroid plexus attachment region in the third ventricle. Scale bars 250 µm.

Supplementary figure 2

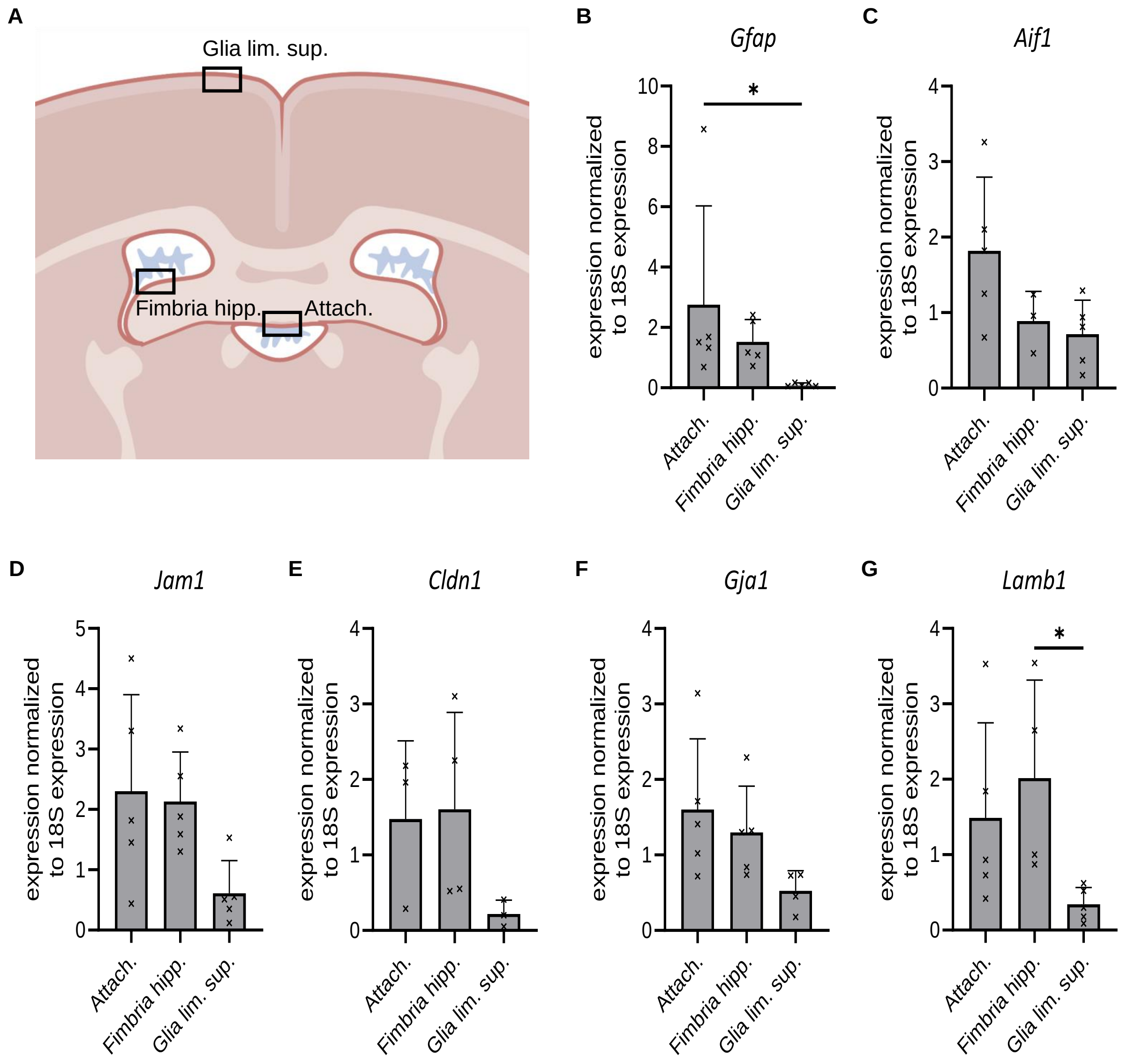

Supplementary Figure 2: Gene expression of glial barrier components at the murine choroid plexus attachment region

(A) Schematic overview of the regions sampled by laser-assisted microdissection. 1: attachment region (Attach.) of the choroid plexus in the third ventricle, 2: dorsal side of the fimbriae hippocampi (Fimbria hipp.), 3: cortical surface with glia limitans superficialis (Glia lim. sup.). (B) to (G) gene expression normalized to of the housekeeping gene 18S. n=5 mice, Data shown as mean and standard deviation. Statistical analysis was performed using Kruskal–Wallis test followed by Dunn's multiple comparisons test: (B) H(2) = 9.437, p = 0.0024 (C) H(2) = 4.378, p = 0.1073 (D) H(2) = 6.080, p = 0.0396 (E) H(2) = 5.109, p = 0.0676 (F) H(2) = 6.209 p = 0.0343 (G) H(2) = 8.086, p = 0.0080 Created with BioRender.com. Method details are given in supplemental material.

Supplementary figure 3

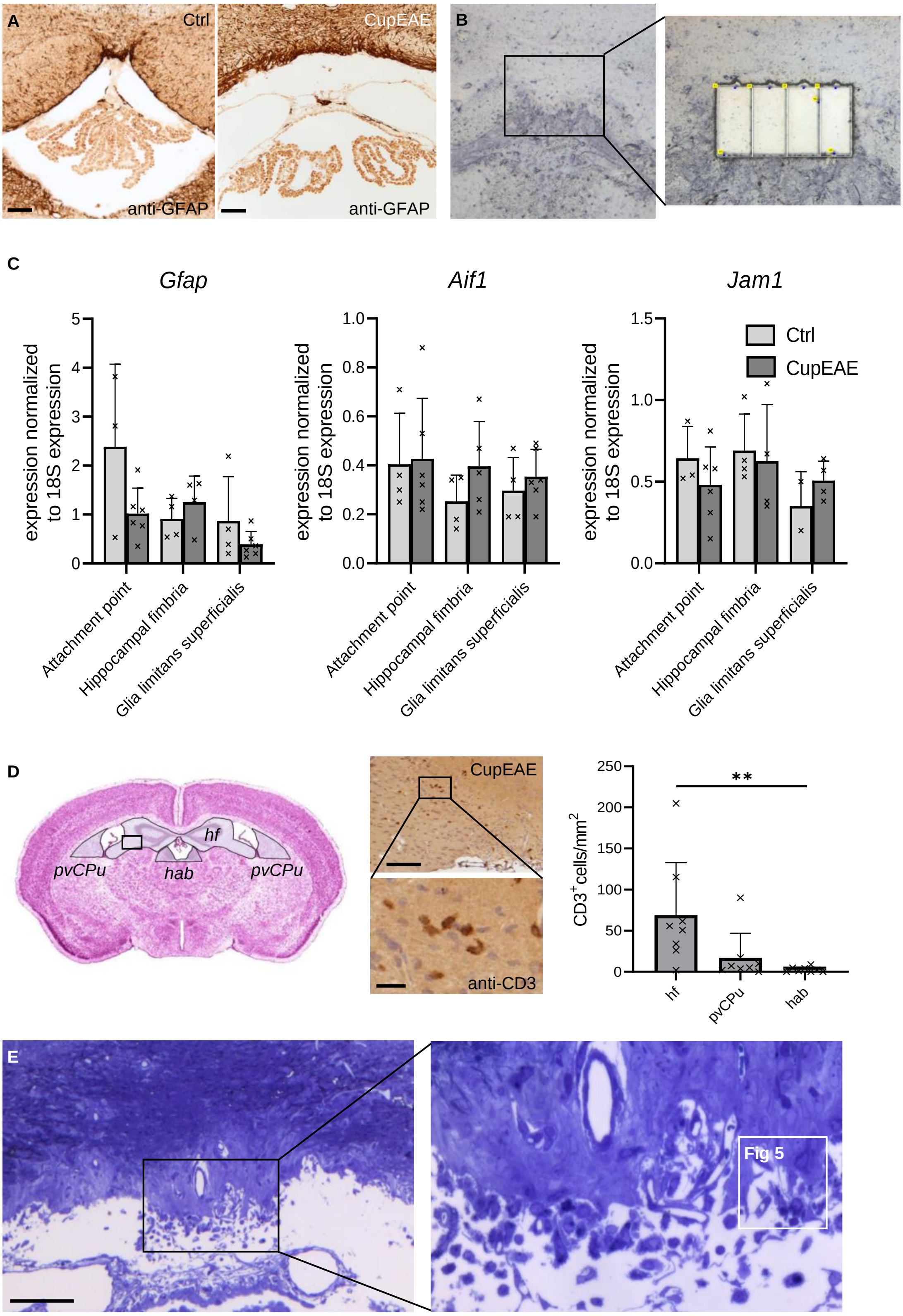

Supplementary Figure 3: Attachment region of the murine third ventricle in the CupEAE model

(A) Representative anti-GFAP labelling of the third ventricle attachment region in control and CupEAE. Scale bar 50 µm. (B) Example of tissue extraction from the attachment region by laser-assisted microdissection. (C) Gene expression of the attachment region, fimbria hippocampi and glia limitans superficialis in control and CupEAE mice. n=4 mice (control), 6 mice (CupEAE)

(D) Quantification of CD3-positive cell density in hippocampal formation (hf) including hippocampal fimbria, periventricular caudate-putamen-complex (pvCPu) and habenulae (hab) with representative anti-CD3 stain from the hippocampal formation. n=8 mice. (E) Semithin section directly adjacent to the region shown in the ultrastructural analysis in Figure 6. The localization of the images shown in Figure 6 is marked in the magnification. Scale bar in (A), (D) and (E) 100 µm, in magnification in (D) 20 µm.

Supplementary Figure 4

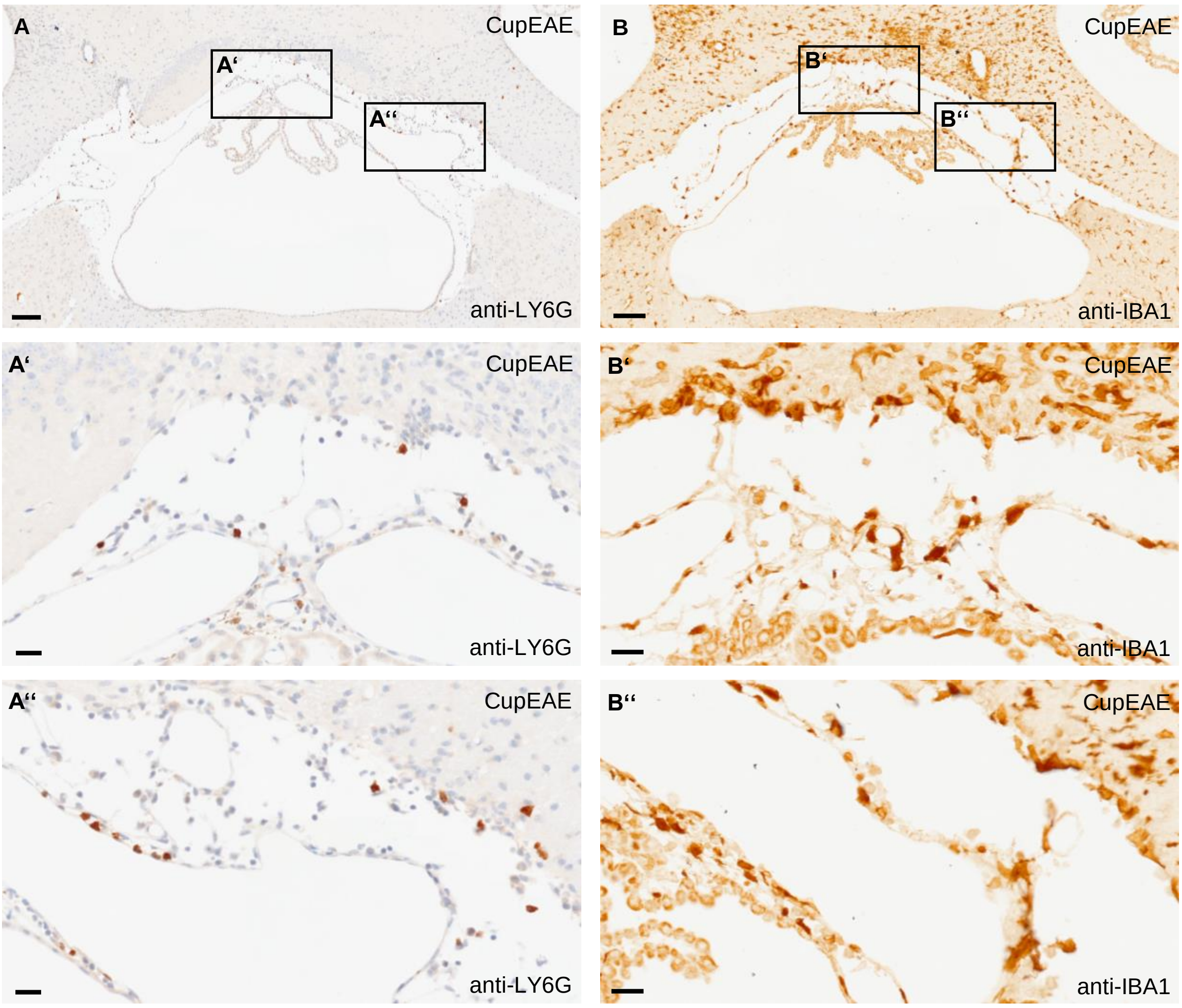

Supplementary Figure 4: Ly6G- and Iba1 immunolabelling in the murine third ventricle in the CupEAE model

(A) Representative anti-Ly6G and (B) anti-Iba1 immunohistochemical stains of the third ventricle in the CupEAE model. Magnifications shown (A’-B’’) are marked by squares. Scale bars in (A) and (B) 100 µm, in (A’-B’’) 20 µm.

Supplementary Figure 5

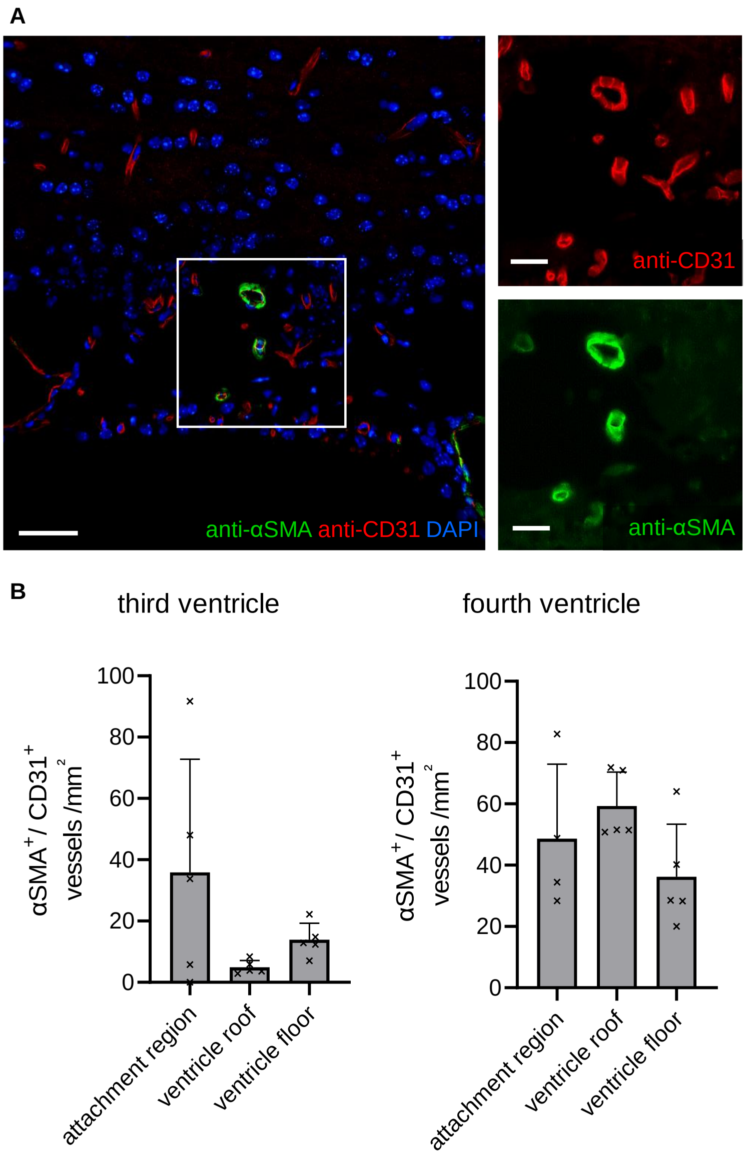

Supplementary Figure 5: Blood vessel density in periventricular regions

(A) Immunofluorescence double staining with anti-CD31 as pan-endothelial cell marker and anti-alpha smooth muscle actin (αSMA) as marker for pre- and postcapillary vessels in the attachment region of the third ventricle. (B) Density of CD31- and αSMA-positive vessels per square millimeter in periventricular regions of the third and fourth ventricle. Please refer to figure 5 for location of the analyzed regions. Scale bars in (A) 50 µm in overview and 10 µm in close ups, n= 5 mice. Statistical analysis was performed using Kruskal–Wallis test followed by Dunn's multiple comparisons test: third ventricle: $H(2) = 4.34$, $p = 0.12$; fourth ventricle: $H(2) = 4.50$, $p = 0.10$

Supplementary material and methods

Laser Capture Microdissection and quantitative PCR

Laser capture microdissection (LCM) with the PALM MicroBeam microscope (Carl Zeiss, Oberkochen, Germany), utilising laser pressure catapult technology was used for the molecular biological investigation of small regions of the mouse brain. The desired tissue area is first outlined manually using the software. The slide is then guided along a fixed laser beam with a wavelength of 355 nm. A laser pulse is then used to catapult the tissue area into the adhesive cap of a specifically designed reaction tube against the force of gravity. The RNeasy Micro Kit (Qiagen, Hilden, Germany) was used for RNA isolation. The eluted RNA concentration was determined in a fluometric manner using the QuantiFluor RNA System (Promega, Mannheim, Germany). When obtaining the attachment regions from murine brains using LCM, very small sample sizes were involved, from which consequently very small amounts of RNA could be isolated. Therefore, reverse transcriptase amplification was performed using Whole Transcriptome Amplification (WTA) technology. The WTA technology is based on two steps. In the first step, RNA is transcribed into complementary DNA (cDNA). In the second step, the cDNA is amplified on the basis of a polymerase chain reaction (PCR) using a WTA polymerase. The procedure was performed following the manufacturer's recommendations (TransPlex® Complete Whole Transcriptom Amplification Kit, Sigma Aldrich, Germany).

Semi-quantitative polymerase chain reaction (qPCR) was performed with SensiMix™ SYBR&Fluorescin kit (Bioline/meridian Bioscience) in the CFX Connect thermocycler (BioRad, Hercules, USA) with the CFX Maestro software. All primers used are summarized below. The analysis was carried out using standard curves and normalization with the housekeeping gene ribosomal 18S. All gene expression data are presented as mean and standard deviation.

Primer for gene expression analysis

Primer	5`-3`
**GFAP**_forward	TCTCGAATGACTCCTCCACTC
**GFAP**_reverse	AAGCTCCGCCTGGTAGACAT
**AIF1**_forward	ATCAACAAGCAATTCCTCGATGA
**AIF1**_reverse	CAGCATTCGCTTCAAGGACATA
**GJA1**_forward	AATTTACTGGGTGGCATCCTAGA
**GJA1**_reverse	GGGAAAGCATCATCGTAACAGAT
**CLDN1**_forward	CTCCTGTCCCCGGAAAACAA
**CLDN1**_reverse	GCAGCAGTTCACAGGCAAAA
**JAM1**_forward	AACTGTAATGGGCACCGAGG
**JAM1**_reverse	TAGGGAGCTGTGATCTGGCT
**LAMB1**_forward	CTAATTCCCTTCTTTGGGCCG
**LAMB1**_reverse	ATGTCGAAGTCACGGAGAGC
**18S**_forward	CGGCTACCACATCCAAGGAA
**18S**_reverse	GCTGGAATTACCGCGGCT
